# Evaluation of the clinical utility of extended non-invasive prenatal testing in the detection of chromosomal aneuploidy and microdeletion/microduplication

**DOI:** 10.1186/s40001-023-01285-2

**Published:** 2023-08-30

**Authors:** Weifang Tian, Yangyang Yuan, Erfeng Yuan, Linlin Zhang, Ling Liu, Ying Li, Jing Guo, Xueyin Cui, Pengyun Li, Shihong Cui

**Affiliations:** 1 Prenatal Diagnosis Center, The Third Affiliated Hospital of Zhengzhou University, Maternal and Child Health Hospital of Henan Province, No. 7 Front Kangfu Street, Er’qi District, Zhengzhou, 450052 China; 2 Department of Medical Research Center, The Third Affiliated Hospital of Zhengzhou University, Maternal and Child Health Hospital of Henan Province, Zhengzhou, 450052 China; 3 Department of Clinical Laboratory Science, The Third Affiliated Hospital of Zhengzhou University, Maternal and Child Health Hospital of Henan Province, Zhengzhou, 450052 China; 4 Perinatal Disease and Prevention of Birth Defects, Advanced Medical Center, Zhengzhou University, Zhengzhou, 450052 China; 5 Henan Provincial Clinical Research Center for Perinatal Medicine, The Third Affiliated Hospital of Zhengzhou University, Zhengzhou, 450052 Henan China

**Keywords:** NIPT-PLUS, Chromosomal microarray analysis (CMA), Rare chromosomal trisomy (RAT), Sex chromosome aneuploidy (SCA), Microdeletion/microduplication (MMS)

## Abstract

**Background:**

With the development of whole-genome sequencing technology, non-invasive prenatal testing (NIPT) has been applied gradually to screen chromosomal microdeletions and microduplications that cannot be detected by traditional karyotyping. However, in NIPT, some false positives and false negatives occur. This study aimed to investigate the applicability of extended NIPT (NIPT-PLUS) in the detection of chromosomal aneuploidy and microdeletion/microduplication syndrome (MMS).

**Methods:**

A total of 452 pregnancies that underwent prenatal diagnostic testing (amniocentesis or chorionic villus sampling) by chromosomal microarray analysis (CMA), were screened by NIPT-PLUS from the peripheral blood sample of the pregnant women. The results of the two tested items were compared and analysed.

**Results:**

Of the 452 cases, 335 (74.12%) had positive CMA results, and 117 (25.88%) had no abnormal results. A total of 86 cases of trisomy 21, 18 and 13 and sex chromosome aneuploidy (SCA) were detected by CMA and NIPT-PLUS, with a detection rate of 96.51% (83/86). Among them, the detection rates of T18, T13; 47, XXY; 47, XXX and 47 XYY were 100%, and the detection rates of T21 and 45 XO were 96.55% and 90%, respectively. The detection sensitivity of rare chromosomal trisomy (RAT) was 80% (4/5). The positive predictive values of NIPT-PLUS for chromosome aneuploidy T21, T18 and T13 and for SCA and RAT were 90.32%, 87.50%, 25.00%, 88.89% and 50%, respectively. A total of 249 cases (74.32%) of chromosomal MMS were detected by CMA. The detection rate of NIPT-PLUS was 63.86% (159/249), and 90 cases (36.14%) were missed. The larger the MMS fragment, the higher the NIPT-PLUS detection sensitivity. In addition, most small fragments were of maternal origin.

**Conclusion:**

The comparison between the CMA and NIPT-PLUS techniques shows that NIPT-PLUS has high sensitivity for detecting chromosomal aneuploidy and chromosomal copy number variations (CNVs) with fragments > 5 M. However, the sensitivity of CNV for fragments < 5 M is low, and the missed detection rate is high. Additionally, confined placental mosaicism and foetal mosaicism are the key factors causing false negatives in NIPT-PLUS, while maternal chromosomal abnormalities and confined placental mosaicism are key contributors to false positives, so appropriate genetic counselling is especially important for pregnant women before and after NIPT-PLUS testing.

## Introduction

Birth defects are the main cause of neonatal deaths and disabilities due mainly to congenital structural abnormalities or genetic diseases, leading to severe clinical symptoms or complications [1]. Among these, 80% of birth defects are caused by genetic factors alone or in synergy [2]. For example, trisomy 21, 18 and 13 syndromes, especially trisomy 21 (Down syndrome), are the most common chromosomal abnormalities found in neonatal birth defects. Some children with trisomy 21 have no obvious deformity phenotype in prenatal ultrasonography and cannot be screened effectively by the technique. Although screening for Down syndrome is now free and widely available to the public, there remains a high rate of missed detection [3]. Sex chromosome aneuploidy (SCA) is a relatively common SCA disorder with twice the incidence of trisomy 21, affecting approximately 1 in 4000 newborns. In addition, chromosomal copy number variation (CNV) is an important factor affecting human disease and phenotypic variation [4]. Some CNVs can lead to severe microdeletion and microduplication syndrome (MMS) [5], while some MMSs are even more likely to occur than Down syndrome. Recent studies have shown that the incidence of MMS in the foetuses of chromosomally normal pregnant women is as high as 1–1.7% [6]. For example, Williams syndrome, DiGeorge syndrome and Prader–Willi syndrome are common high-incidence syndromes. In addition, MMS causes about 12% of unexplained intellectual disabilities, various deformities and developmental delays [7].

Effective prenatal screening and prenatal diagnosis techniques are particularly important for preventing birth defects. Cytogenetic karyotype analysis has always been used as the gold standard for prenatal diagnosis. It allows the visual observation of abnormalities in terms of chromosome number and large fragment structure, but it cannot fully detect microdeletions, microduplications or uniparental diploidy. As the two main CNV detection techniques, chromosomal microarray analysis (CMA) and low-depth CNV sequencing provide high-resolution analysis of microdeletions and microduplications [8, 9]. In particular, CMA includes array-based comparative genomic hybridisation and a single-nucleotide polymorphism (SNP) array, which is a high-resolution genome-wide screening technology that can be used to analyse both chromosomal aneuploidy and CNVs [10]. Chromosomal array analysis has become the main molecular detection technology in addition to karyotyping. Chromosomal microarray analysis (CMA) as a diagnostic technique.

With the development of next-generation sequencing technology, non-invasive prenatal testing (NIPT) technology based on cell-free foetal DNA (cffDNA) in maternal peripheral blood has been shown to outperform traditional screening methods and is rapidly becoming the primary screening test in clinical practice [11]. This method has been validated in multiple clinical cohorts, confirming that NIPT is highly sensitive and specific for T13, T18 and T21 aneuploidy risk screening [12]. Other chromosomal abnormalities can be revealed by the low-coverage whole-genome sequencing of maternal and placental DNA fragments [13]. The antenatal extended NIPT (NIPT-PLUS) technique is gradually being applied clinically. Compared with NIPT detection technology, NIPT-PLUS increases the output of sequencing data. However, due to the low incidence of individuals with chromosomal abnormalities and the use of sequencing platforms, data analysis parameters and types of chromosomal abnormalities in clinical units, the accuracy of NIPT-PLUS for the detection of RATs and CNVs is still unclear, limiting its wide clinical application. Extended NIPT (NIPT-PLUS) technique as a screening technique.

In recent years, most research has focused on retrospective studies of large-sample NIPT assays, which focus on positive predictive values (PPVs) and accuracy analyses of aneuploidy. However, few studies have analysed the detection rate and missed detection rate of NIPT-PLUS for detecting chromosomal aneuploidy and CNV. In this study, we compared 452 pregnant women with a prenatal diagnosis who underwent CMA and NIPT-PLUS to compare and analyse the results of the two testing methods. The detection rate and optimal detection range of NIPT-PLUS for common chromosomal aneuploidy, SCA, RAT and MMS were evaluated, respectively. Simultaneously, the key factors causing false-negative and false-positive NIPT results were analysed, and the clinical utility of the method was evaluated further.

## Materials and methods

### Study subjects and sample types

**Fig. 1:**
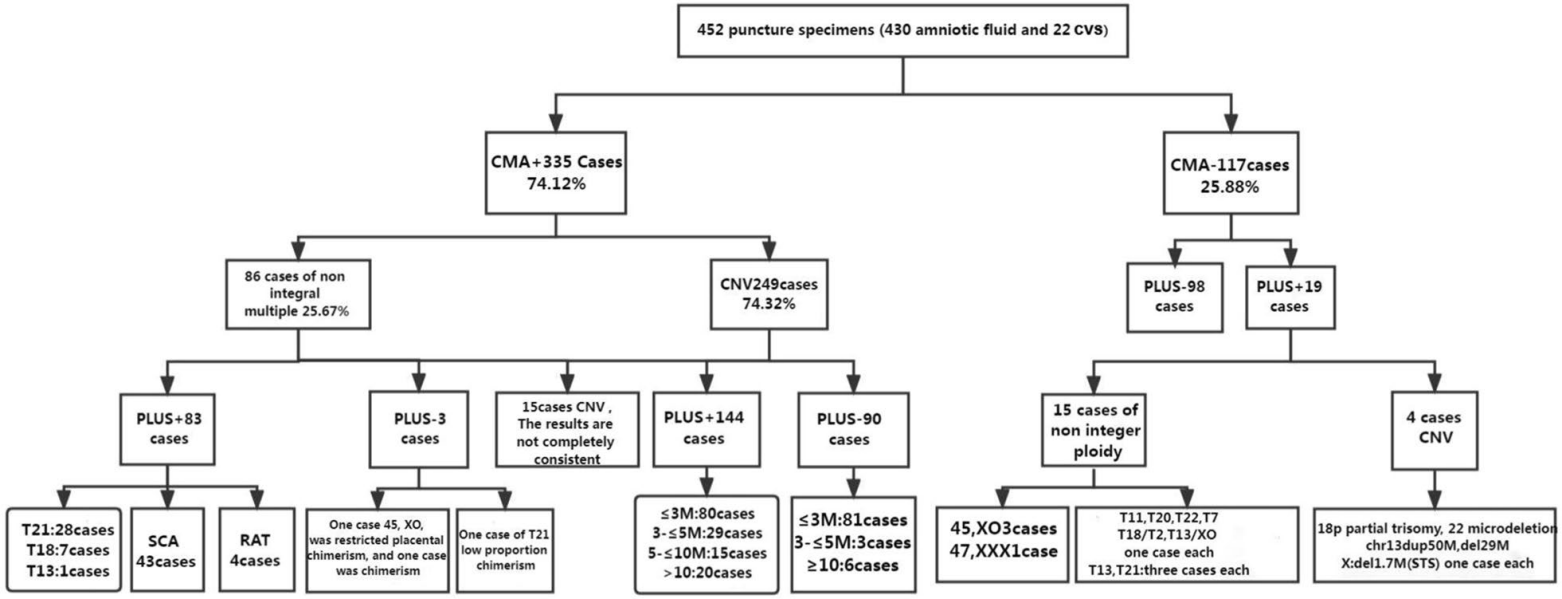
Summary of CMV results and NIPT-PLUS results of 452 prenatal samples

This study recruited 452 pregnant women with indications for a prenatal diagnosis from November 2017 to June 2019 in the Third Affiliated Hospital of Zhengzhou University (Fig. 1). Inclusion criteria Selection of pregnant women with prenatal diagnosis indications (such as advanced maternal age, abnormal Down syndrome screening results, abnormal ultrasound examination structure and other indications). Exclusion criteria: (1) gestational age < 12 weeks, multiple births (> 2); (2) couples with clear chromosomal abnormalities; (3) pregnant women received allogeneic blood transfusion within 1 year; (4) pregnant women received immunotherapy, stem cell therapy, transplantation within 4 weeks; (5) pregnancy with malignant tumors; (6) family history of genetic diseases or fetal high-risk genetic diseases and chromosomal diseases.

Each subject underwent prenatal genetic counselling and prenatal diagnostics [amniocentesis in 430 cases and chorionic villus sampling (CVS) in 22 cases] and CMA testing of their amniotic fluid or CVS. At the same time, the peripheral blood of the pregnant women was drawn for the identification of maternal blood contamination and NIPT-PLUS detection before the experiment. Before enrolment in the study, each pregnant woman gave detailed information and provided written informed consent.

### Sample collection

Under aseptic conditions, amniocentesis or chorionic villus sampling was performed under ultrasound by a physician with prenatal diagnosis qualifications, and 8–15 ml of amniotic fluid or chorionic villus samples (CVS) was extracted. In addition, 5–10 ml of the pregnant women’s peripheral blood was drawn via an EDTA anticoagulation tube for the identification of maternal blood contamination and NIPT-PLUS detection.

### CMA detection and data analysis

Amniocytes were collected by centrifugation, villous cells were collected by digestion, and DNA was extracted using a QIAamp blood DNA purification kit (QIAGEN, Germany). Maternal blood contamination was identified by short tandem repeat locus alignment. After confirming that there was no maternal blood contamination, a CytoScan 750K chip (Affymetrix, USA) was used to detect genome CNVs. The specific experimental procedures, including enzyme digestion, ligation, PCR amplification, purification, quantification, fragmentation, labelling, chip hybridisation, washing and scanning, were performed with reference to the published literature [14]. A raw data analysis was performed using CHAS software (version 4.2.0.80). According to the guidelines of the American Society for Medical Genetics and Genomics, CNVs ≥ 100 kb were interpreted and their data analysed [15, 16].

### NIPT-PLUS detection and data analysis

The peripheral serum supernatant of the pregnant women was collected by centrifugation at 1600×*g* for 10 min. Three aliquots were transferred into cell-free DNA tubes; two were stored at − 80 °C for later use, and one was subjected to DNA extraction, library construction, emulsion PCR and onboard sequencing in accordance with published studies [17]. Sequencing data were analysed using an ion semiconductor sequencing platform and software. To assess the foetal risk of aneuploidy disease, samples with an absolute chromosome Z score of ≥ 3 were classified as positive. Chromosomal microdeletions and microduplications were analysed by comparison with the human genome reference sequence (the GRCh37 sequence). The reference genome was divided into 300,000 sliding windows containing the same number of reads, and the relative number of reads in this window was defined as the ratio of the number of reads in the same window to the average number of reads. The linear relationship between the GC content and the relative number of reads was analysed using the least-squares method. The types of foetal chromosomal abnormalities were predicted using the dynamic threshold method and a quadratic segmentation algorithm.

Methods for assessing the proportion of cffDNA [18]: (1) Y-chromosome estimation: the karyotype of healthy women is 46, XX, which does not contain the Y chromosome, that is to say, cffDNA is detected once cffDNA fragments derived from the Y chromosome are detected in maternal plasma. (2) SeqFF estimation: cffDNA detected in maternal plasma is not uniformly and sporadically distributed in all regions of the chromosome. In fact, in some chromosomal regions, cffDNA accounts for a high proportion, while in some chromosomal regions, cffDNA accounts for a small proportion. Using this characteristic of cffDNA, each autosome was divided into interval windows (bins) every 50 kb, and bins with cffDNA fragments of 100 to 150 bp were selected to calculate the proportion of bins with shorter cffDNA fragment lengths to all bins, and a high regression model, the seqFF model, was established using GC content, read number and other indicators as parameters, and the cffDNA proportion could be estimated by this model. In this study, Y chromosome estimation and seqFF estimation were combined to assess the proportion of cffDNA in specimens. 3.5% was selected as the lowest detection limit for the proportion of cffDNA.

### Statistical analysis

The Python open-source tool (https://www.python.org/) was used for data drawing and statistical analysis, statistical distributions of clinical indications of the enrolled samples, calculations of the mean and maximum and minimum values of NIPT-PLUS quality control data and the statistical analysis of detection rate, sensitivity and PPV.

## Results

### Clinical features of the samples

Among the prenatal diagnosis samples, there were 430 cases of amniotic fluid and 22 cases of CVS. The average age of the pregnant women was 29.5 years, majority was between 19 and 34 years (376, 83.19%), and 72 cases of advanced maternal age, accounting for 15.93% (≥ 35 years). The gestational week was 160 (82–255) days, of which the second trimester accounted for the highest proportion (85.40%). Among the clinical indications of the samples, 286 cases (63.27%) of ultrasound abnormalities accounted for the majority, followed by a high risk of Down syndrome (14.16%) on the combined first trimester screening and abnormal NIPT results (13.05%). Within the NIPT-PLUS sequencing quality control results, the average foetal concentration (i.e. the cffDNA%) was 23.32%, the average effective data volume was 8.5 Mb, and the average read length of the sequenced short fragments was 135 bp (see Table 1).

**Table 1:** Clinical characteristics and sequencing parameters of the 452 enrolled samples

Clinical features	Data
Amniotic fluid	430 (95.13%)
CVS	22 (4.87%)
Maternal age (years)	29.5 (15–47)
≤ 18	4 (0.88%)
19–34	376 (83.19%)
≥ 35	72 (15.93%)
Gestational week (days)	160 (82–255)
≤ 84	3 (0.66%)
> 85 to ≤ 196	386 (85.40%)
> 197	63 (13.94%)
CVS gestational week (days)	90.77 (82–98)
Amniotic fluid gestational week (days)	164 (105–225)
Clinical diagnosis	
Abnormal NIPT	59 (13.05%)
Adverse pregnancy history	22 (4.87%)
Ultrasound abnormalities	286 (63.27%)
Advanced maternal age (≥ 35)	72 (15.93%)
One of the parents has chromosomal abnormalities (two of them are couples with chromosomal abnormalities for three generations of test tubes)	8 (1.77%)
High risk of Down’s syndrome screening	64 (14.16%)
Sequencing quality control	
cffDNA concentration (%) of chorionic villus sampling	20.53 (11.86–46.44)
cffDNA concentration (%) of amniocentesis	23.51 (4.20–88.28)
Uniquely mapped reads (Mb)	8.5 (6.3–21.5)
Average read length of the sequencing of short fragments (bp)	135 (112–156)

*NIPT* noninvasive prenatal testing, *cffDNA* cell-free fetal DNA

### Sensitivity and PPV of NIPT-PLUS for aneuploidy detection

Extended NIPT of the maternal peripheral blood was performed on 452 prenatal samples for CMA detection; 335 cases were positive for CMA, with 117 cases showing no abnormal results. The positive rate of the included samples was 74.12%. Among the 335 CMA-positive samples, 86 (25.37%) had abnormal results for aneuploidy, of which 83 were detected by NIPT-PLUS, with a sensitivity of 96.51% (83/86).

Among the 37 common trisomy cases (T21, T18 and T13), 36 cases were detected by NIPT-PLUS, including 28 cases of T21, 7 cases of T18 and 1 case of T13. The sensitivities of NIPT-PLUS for common trisomy detection were 96.55%, 100% and 100%, respectively. One case of T21 detected by CMA was missed by NIPT-PLUS, with the CMA result suggesting chimeric T21. Further fluorescence in situ hybridisation (FISH) confirmed low-level mosaicism of about 13.67% (Table 2). In 1 case of T18, CMA and NIPT-PLUS detected 47, XXY syndrome at the same time.

**Table 2:** Aneuploidy detected by NIPT-PLUS

	Aneuploidy	Results of CMA	Results of NIPT-PLUS	Case	Remark	Sensitivity 95% CI
Common trisomy	T21	arr[GRCh37] 21q11.2q22.3(15,016,486_48,093,361)×3	T21	28		96.55% (80.37–99.82%)
		arr[GRCh37] 21q21.3q22.3(28,054,301_48,093,361)×2–3	No abnormalities	1	FISH:47,XN,+21[19]/46,XN[140](13.57%)	
	T18	arr[GRCh37]18p11.32q23(136,227_78,013,728)×3	T18	6		100% (56.00–100.00%)
		arr[GRCh37]18p11.32q23(136,227_78,013,728)×3 arr[GRCh37] Xp22.33q28(168,551_155,233,098)×2	T18 47,XXY	1		
	T13	arr[GRCh37]13q12.2q32.3(28,722,472_100,577,274)×3	T13	1		100% (54.62–100.00%)
SCA	45,XO	arr[GRCh37] Xp22.33q28(168,551_155,233,098)×1	45,XO	12		90.90% (69.37–98.40%)
		arr[GRCh37] Xp22.33q28(168,551_155,233,098)×1	45,XO chr4:del:177M188M size = 12.00 M	1	Repeat No. 4 12 Mb false positive	
		arr[GRCh37] Xp22.33q22.1(168,551_98,064,447)×1–2 arr[GRCh37] Xq22.1q28(98,064,447_155,233,098)×1	45,XO	1	Karyotype: 46,X,i(Xq)[5]/45,X	
		arr[GRCh37] Xp22.33q28(168,551_155,233,098)×1_2	Chimeric 45, XO (cffDNA: 21.04%, chrX_z_score: _11.52)	1	Karyotype: 45,X[4]/46,XX [16](20%)	
		arr[GRCh37]Xq23q28(110,557,268_155,233,098)×1	45,XO	1	Karyotype: 46,X,del(Xq)	
		arr[GRCh37]9p24.3q13(208,454_68,342,770)×3 arr[GRCh37]Yp11.2q11.23(5,997,807_28,799,653)×0	45,XO chr9:dup:0M_37M size = 38.00 M	1		
		arr[GRCh37]Xp22.33q28(168,551_155,233,098)×1–3	45,XO	1	Karyotype: 46,X,der(X)(qter→q13::p22.1→qter)	
		arr[GRCh37] Xp22.33q28(168,551_155,233,098)×1	No abnormalities	1	MLPA verifies as restricted placental mosaicism	
		arr[GRCh37] Xp22.33q28(168,551_155,233,098)×1_2	No abnormalities	1	False negative	
	47,XXY/T20^a^	arr[GRCh37] Xp22.33q28(168,551_155,233,098)×2	47,XXY	10		100% (65.54–100.00%)
		arr[GRCh37]10q25.3q26.12(116,585,858_122,530,451)×1 arr[GRCh37] Xp22.33q28(168,551_155,233,098)×2	47.XXY	1	Chr10 missing 5.94 Mb missed detection	
		arr[GRCh37]Xp22.33q28(168,551_155,233,098)×2 arr[GRCh37]20p13q13.33(61,661_62,913,645)×2–3	47,XXY	1	T20 missing detection*	
	47,XXX	arr[GRCh37] Xp22.33q28(168,551_155,233,098)×3	47,XXX	7		100% (59.77–100.00%)
		arr[GRCh37] Xp22.33q28(168,551_155,233,098)×3	47,XXX/T5	1	T5 false positive	
	47,XYY	arr[GRCh37] Yp11.31p11.2(2,650,424_9,172,827)×2 arr[GRCh37] Yp11.2q11.23(9,329,329_28,799,654)×2	47,XXY	3		100% (46.29–100.00%)
			45,XO	2		
RAT	T2	arr[GRCh37]] 2p25.3q37.3(12,770_242,524,587)×2–3	T2	1		80% (53.65–99.12%)
	T7	arr[GRCh37] 7p22.3q36.3(43,376_159,119,707)×2–3	T7	1		
	T16	arr[GRCh37]16p13.3q24.3(85,880_90,155,062)×2–3	T16	1		
	T22	arr[GRCh37]22q11.1q13.33(16,888,899_51,197,766)×2–3	T22	1		
	47,XXY/T20^a^	arr[GRCh37]Xp22.33q28(168,551_155,233,098)×2 arr[GRCh37]20p13q13.33(61,661_62,913,645)×2–3	47,XXY	1	T20 missed in this case	

“*” means the specific missing fragment is not detected; “a” is a same case

*CMA* chromosomal microarray, *NIPT-PLUS* non-invasive prenatal testing-plus, *SCA* sex chromosome aneuploidy, *RAT* rare chromosomal trisomy, *MLPA* multiplex ligation-dependent probe amplification, *CI* confidence interval

The CMA results indicated 45 cases of SCA, of which 43 were detected by NIPT-PLUS, with a sensitivity of 95.55%. The CMA results revealed 45, XO in 20 cases, while NIPT-PLUS detected 18 cases; therefore, the sensitivity was 90%. In 12 cases, the NIPT-PLUS detection result was completely consistent with that of CMA, which was 45, XO. In 1 case with a missing short arm of X (copy number 1–2) and the complete absence of the long arm (46, X, del [Xq]), NIPT-PLUS indicated that it was 45, XO, and the final karyotype result indicated that it was 45, X/46, delX (p) (q22.1). One case was identified by both CMA and NIPT-PLUS as chimeric, and the chimeric ratio of karyotype verification was about 20%. In 1 case, the results of both the CMA and NIPT-PLUS techniques were suggestive of 45, XO, and the result of karyotype verification was 46, X, der (X) (qter→q13::p22.1→qter). In addition to 45, XO, CMA and NIPT-PLUS detected a false positive of 9p24.3q13 repeat 38 Mb in a case unclear NIPT-PLUS suggested 45,X and CMA detected 9p24.3q13 repeat 38 Mb Furthermore, NIPT-PLUS missed the detection of 2 cases of 45, XO. One case was obtained from the placenta of a woman in whom labour was induced, and multiplex ligation-dependent probe amplification (MLPA) confirmed the presence of confined placental mosaicism. One case of CMA suggested that it was chimeric 45, XO (Table 2).

A total of 12 cases of 47, XXY were detected by CMA, and all were detected by NIPT-PLUS. Among these, 2 cases of 10q25.3q26.12 deletion 5.94 Mb and chimeric T20 detected by CMA were missed by NIPT-PLUS. Eight cases of 47, XXX were detected by NIPT-PLUS, and 1 case was additionally detected by NIPT-PLUS as a false positive for T5. There were 5 cases of 47, XYY; 3 cases were consistent with the NIPT-PLUS results, while the other 2 cases had NIPT-PLUS results of 45, XO (Table 2).

In total, CMA detected 5 cases of RAT, which were chimeric T22, T16, T2 and T7 and the above-mentioned chimeric T20/47, XXY. Except for chimeric T20, all were detected by NIPT-PLUS, with an 80% sensitivity for RAT detection.

According to the detection of NIPT-PLUS and the analysis of the CMA diagnosis, the PPVs of NIPT-PLUS for the detection of chromosomal trisomy were as follows: T21 (90.32%), T18 (87.50%), T13 (25.00%), SCA (88.89%) and RAT (50%) (Table 3).

**Table 3:** PPV of aneuploidy detected by NIPT-PLUS

	NIPT-PLUS+	CMA+	PLUS−	PPV%	95% CI
T21	31	28	1	90.32	73.10–97.47%
T18	8	7	0	87.50	46.68–99.34%
T13	4	1	0	25.00	13.19–78.06%
SCA	45	40	1	88.89	75.15–95.84%
RAT	8	4	0	50	17.45–82.55%

*CMA* chromosomal microarray, *NIPT-PLUS* non-invasive prenatal testing-plus, *PPV* positive predictive values, *CI* confidence interval; “+” means “positive”, “−” means “negative”

### Sensitivity of NIPT-PLUS for CNV detection

**Fig. 2:**
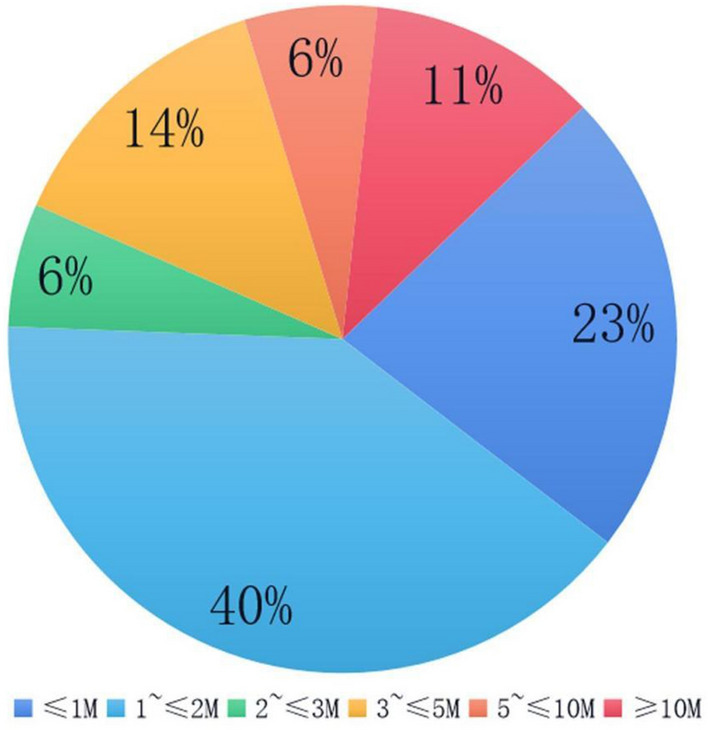
Size distribution of CNV fragments detected by CMA

Among the 335 samples with positive CMA results, 249 cases were suggested to be CNV; of these, 159 cases (63.86%) were detected by NIPT-PLUS, and 144 cases were completely consistent with the CMA results, with a sensitivity of 67.94%. The NIPT-PLUS method did not detect CNVs in 90 cases, meaning that CNV detection was missed in 36.14% of cases. The size distribution of CNV fragments suggested by CMA is shown in Fig. 2. The largest proportion of CNV fragments was between 1 and 2 Mb, accounting for about 40% of the total, followed by fragments between 500 kb and 1 Mb, accounting for 23%.

As shown in Table 4, there were 53 samples with a CNV size of 500 kb to ≤ 1 Mb. Twenty-one samples were detected by NIPT-PLUS, 17 of which were suggested to be from the mother, and 4 samples suggested that the fragment was larger than the actual CNV fragment (about 2 Mb). In all, 32 cases were detected, with a missed detection rate of 60.37%. A total of 94 cases of CNV fragment size between 1 and ≤ 2 Mb were detected, of which 49 cases were detected by NIPT-PLUS, with a sensitivity of 52.13%. However, NIPT-PLUS suggested maternal carriage in 41 cases and a fragment length of > 5 Mb in 2 cases, while it missed 45 cases. The missed detection rate was 47.87%. Among the samples with CNV fragment lengths of 2 to ≤ 3 Mb and 3 to ≤ 5 Mb, NIPT-PLUS detected 10 cases and 29 cases, respectively, of which 14 cases were suggested as being carried by the mother, and 1 case had a suggested fragment length longer than the actual CNV result (Table 4). Four cases and three cases were missed, with missed detection rates of 28.57% and 9.37%, respectively. There were 15 samples with CNV fragment lengths of between 5 and ≤ 10 Mb, all of which were detected by NIPT-PLUS. However, in 2 cases, NIPT-PLUS suggested maternal carriage, and in 1 case, the suggested fragment length was longer than 10 Mb. Among the CNV fragments ≥ 10 Mb, NIPT-PLUS detected 20 cases, none of which suggested maternal carriers, but 6 cases were missed, with a missed detection rate of about 23.08%.

**Table 4:** Analysis of CNV fragment size detection results by NIPT-PLUS

CNV fragment size	CMA+ (case)	NIPT-PLUS+	Tips in NIPT-PLUS + come from the mother	NIPT-PLUS hints that the fragment is larger than the CNV	NIPT-PLUS−	Sensitivity
500 kb to ≤ 1 Mb	53 (22.65%)	21 (39.62%)	17 (80.95%)	4	32 (60.37%)	39.62% (26.76–53.98%)
1 to ≤ 2 Mb	94 (40.17%)	49 (52.13%)	41 (83.68%)	2	45 (47.87%)	52.13% (41.63–62.45%)
2 to ≤ 3 Mb	14 (5.98%)	10 (71.43%)	5 (50%)	0	4 (28.57%)	71.43% (42.00–90.42%)
3 to ≤ 5 Mb	32 (13.68%)	29 (90.63%)	9 (31.03%)	1	3 (9.37%)	90.63% (73.83–97.54%)
5 to ≤ 10 Mb	15 (6.41%)	15 (100%)	2 (13.33%)	1	0	100.00% (74.65–100%)
≥ 10 Mb	26 (11.11%)	20 (76.92%)	0	0	6 (23.08%)	76.92% (55.91–90.25%)
Total	234	144 (61.54%)	74	8	90 (38.46%)	61.54% (54.94–67.74%)

*CNV* chromosomal copy number variation, *CMA* chromosomal microarray, *NIPT-PLUS* non-invasive prenatal testing-plus; “+” means “positive”, “−” means “negative”

**Fig. 3:**
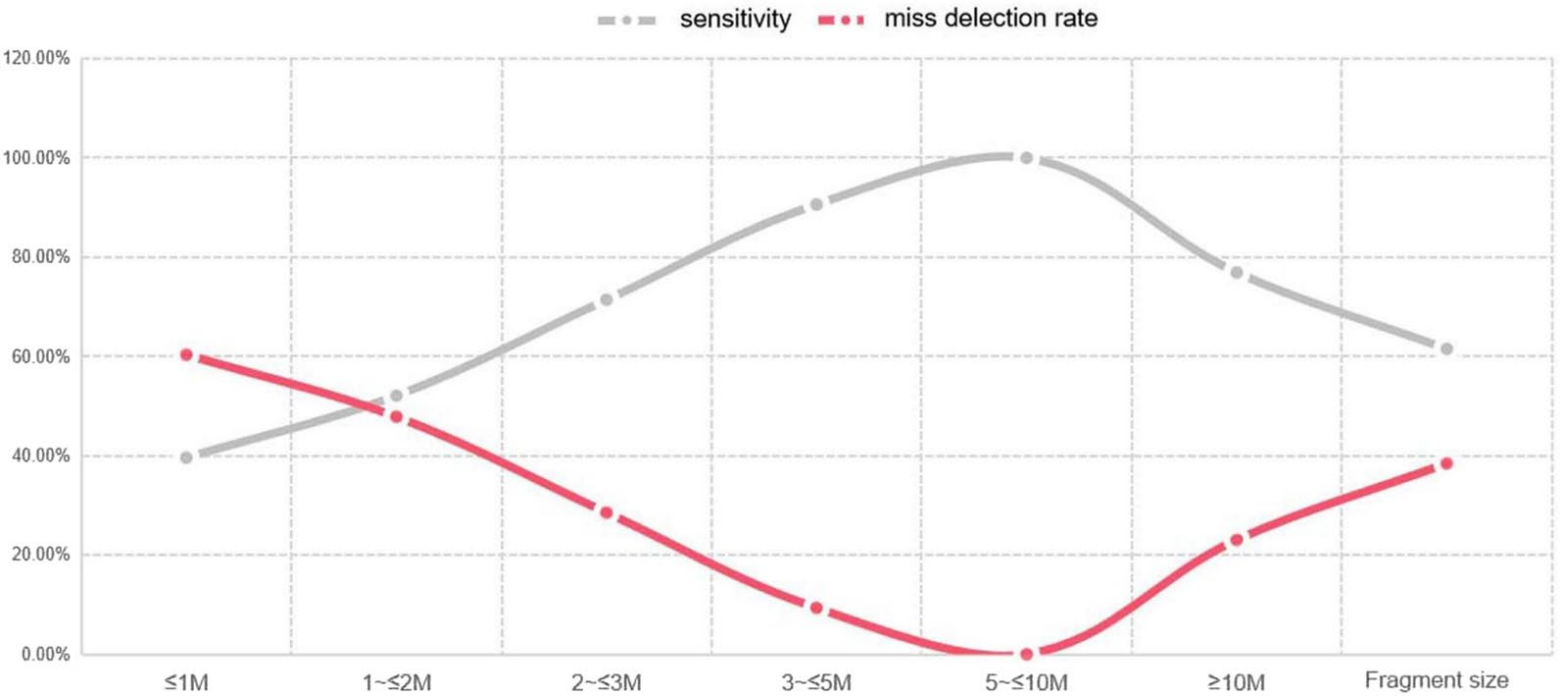
Variation trend of sensitivity and missed detection rate of NIPT-PLUS for detecting different fragment sizes of CNV

According to the graph analysis presented in Fig. 3, the sensitivity of NIPT-PLUS to CNV detection increased with the increase in CNV fragments, the sensitivity of CNV for the fragments between 5 and 10 Mb was the highest, while the sensitivity of CNV for the fragments > 10 Mb decreased.

When analysing the detection of common syndromes by CMA, NIPT-PLUS had a sensitivity of 100% for the detection of 1p36 microdeletion, cri-du-chat syndrome and chromosome 9p deletion (duplication) syndrome. In this study, the fragment sizes of these three syndromes were all larger than 5 Mb. The sensitivity of the detection of DiGeorge syndrome reached 92.31%, the sensitivity of renal cysts and diabetes syndrome (RACD) and Angelman/Prader–Willi syndrome was 73.33% and 66.60%, respectively, and the sensitivity of Williams–Beuren syndrome was the lowest, at about 33.3% (Table 5). As shown in Fig. 3, the smaller the fragment, the lower the sensitivity of NIPT-PLUS detection and the greater the possibility of missed detection.

**Table 5:** Sensitivity of NIPT-PLUS for detecting common syndromes

Common syndrome	Case	Detect fragment size (Mb)	NIPT-PLUS+	Sensitivity (%)	Missing detection rate
1p36 microdeletion	2	5–10	2	100	0
5p15.2-13.3 (cri du cat syndrome)	2	> 10	2	100	0
Williams–Beuren syndrome	3	1–2	1	33.30	66.70%
Chromosome 9p deletion (duplication) syndrome	4	> 10	4	100	0
Angelman/Prader–Willi syndrome	3	3–5	2	66.70	33.30%
Renal cysts and diabetes (RCAD) syndrome	15	< 2	11 (8 cases suggested maternal origin)	73.33	26.67%
22q11.2 deletion syndrome (DiGeorge syndrome)	13	3	12	92.31	7.69%

*RCAD* renal cysts and diabetes, *NIPT-PLUS* non-invasive prenatal testing-plus

In another 15 CMA-detected CNV samples, the NIPT-PLUS detection results were not completely consistent. Among them, 4 cases of CNV fragments were also detected as false positives for aneuploidy (T7, T9, 47, XXX, 47, XYY, respectively) and 6 cases of large-fragment CNV also had false positives. In 3 cases, CMA detected the deletion and duplication of two fragments, while NIPT-PLUS detected only one of them, and all the missed fragments were < 3 Mb in size. The CMA result of another sample suggested regions of homozygosity (ROH) with two large fragments of chromosome 16, but the NIPT-PLUS result suggested complete trisomy 16. In 1 sample, the CMA result indicated that there was an 18 Mb duplication of chromosome 3, but the NIPT-PLUS sample indicated that there were multiple other chromosomal abnormalities in addition to the duplication of the entire chromosome 3.

### False positives of NIPT-PLUS

Among the 117 cases with no abnormality indicated by the CMA results, 98 cases were indicated as low risk by the NIPT-PLUS results. The NIPT-PLUS technique produced 19 false positives for chromosomal abnormalities. Among them were 15 false-positive cases of aneuploidy, 3 cases of 45, XO; 1 case of 47, XXX; 3 cases of T21 and 3 cases of T13. The cases of RAT included T11, T20, T22, T7, T18/T2 and a case of T13/XO. There were another 4 cases of CNV false positives, including 1 case of 18p partial trisomy, 1 case of 22q11.2 microdeletion, 1 case of chromosome 13 duplication of 50-Mb and 29-Mb deletion and 1 case of X chromosome p22.31 region 1.7-Mb deletion (STS area), but the NIPT-PLUS tip was from the mother.

## Discussion

Most retrospective research articles have mainly analysed the PPVs of NIPT-PLUS for aneuploidy and CNV, and few have analysed the sensitivity and miss rate of NIPT-PLUS for aneuploidy and CNV. The sensitivity and miss rates are important to enable pregnant women to correctly understand this means of testing and genetic counselling. Our data show that through the parallel detection of CMA and NIPT-PLUS, the optimal appropriate range for NIPT-PLUS detection and the sensitivity and miss rate for CNV detection can be obtained. A study by Maya et al. [19] found that the residual risk of clinically significant CNVs in pregnancies without structural sonographic anomalies is appreciable and depends on the extent of non-invasive prenatal screening and maternal age. However, it is not clear what the detection rate of NIPT-PLUS is in pregnant women with indications for a prenatal diagnosis, such as ultrasound abnormalities. In our study, the selected samples were from pregnant women with indications for a prenatal diagnosis. Indications for a prenatal diagnosis were advanced maternal age (15.93%), abnormal Down syndrome screening results (14.16%), structural anomalies on ultrasonography (63.27%) and other indications (13.05%).

Studies have shown that compared with traditional screening methods, NIPT-PLUS has higher sensitivity and specificity in detecting common foetal chromosomal aneuploidies [20]. The PPV of T21, T18 and T13 screening was 80–94%, 60–85% and 20–63%, respectively [21, 22]. This study did not conduct a retrospective analysis but conducted a direct prenatal diagnosis based on indications for a prenatal diagnosis. This is not the same advantage as in the other studies. Pregnant women who chose CMA testing were tested directly by NIPT-PLUS to evaluate the detection efficiency of the technique for chromosomal aneuploidy and CNVs. Nevertheless, the analysis of NIPT-PLUS data from this study showed that the PPVs of NIPT-PLUS for target chromosomal aneuploidy T21, T18 and T13 were 90.32%, 87.50% and 25.00%, respectively. This is consistent with the PPVs of common trisomy reported in other studies. The detection rates of T21, T18 and T13 were 96.55%, 100% and 100%, respectively, which was also consistent with the rates in the published literature [23].

The literature reports that the PPV of NIPT-PLUS for SCA and MMS is not as high as that for commonly seen T21, T18 and T13 [24, 25]. Studies have shown that the PPV of SCA detected by NIPT-PLUS is about 46.7% [26]. The data of this study showed that the PPV of SCA detected by NIPT-PLUS was 88.89%, which could be related to the fact that this study was not a large-scale retrospective study but rather, it observed a specific obstetric population. However, this study provides good reference data for the detection rate of SCA by NIPT-PLUS. From our data, it can be seen that the sensitivity of NIPT-PLUS to 47, XXY; 47, XXX and 47, XYY was 100%, but the sensitivity to 45, XO was only 90%, which is also consistent with the conclusions reported elsewhere, i.e. NIPT-PLUS can detect XXX, XXY and XYY better than 45, XO [22, 27]. Moreover, the comparison with the results of the prenatal diagnoses shows that NIPT-PLUS could not accurately indicate partial mosaicism 45, XO; partial X chromosome deletion or the X isoarm chromosome, although it could be used as a first-line screening technology, which is enough to prompt pregnant women and clinicians to consider invasive prenatal diagnosis. By analysing the case of 45, XO, it can also be seen that CMA technology has disadvantages in detecting low-ratio mosaicism and chromosomal translocation. The FISH technique and cytogenetic karyotyping are better for detecting chimerism, and cytogenetic karyotyping has an irreplaceable role in detecting chromosomal translocation. Therefore, for abnormal results of aneuploidy detected by NIPT-PLUS, in addition to a molecular-level diagnosis, cytogenetic karyotype analysis may be also an indispensable test.

Furthermore, RAT is also relatively common in the NIPT-PLUS test, but most of the time it is considered to be a false-positive result. According to the genome content on the chromosome, the types of RAT mainly reported by NIPT-PLUS prenatal screening in our laboratory were T9, T15, T16, T20 and T22. According to the present study’s data, the sensitivity of NIPT-PLUS for RAT detection reached as high as 80%, but there were many false positives, and the PPV was only 50%. This may be related to the non-invasive false positives caused by confined placental mosaicism [28]. The cffDNA in maternal peripheral blood detected by NIPT-PLUS is believed to be mainly from the apoptosis of placental trophoblast cells [29], which cannot fully represent the foetus. The chromosomal abnormality that occurs only in the placenta and not in the foetus is called restricted placental mosaicism [30], and studies have shown that its incidence is about 1–2% [31]. Because it is often assumed that foetuses with rare chromosomal trisomy are generally miscarried or stop developing at an early stage, there are insufficient data to support the detection rate and PPV of RAT using the NIPT-PLUS technique.

Compared with ordinary NIPT, NIPT-PLUS increases the volume of effective data and can detect more microdeletions and microduplications [32]. The literature shows that the PPV of NIPT-PLUS for CNV is 60–100% [33], and the latest literature shows that the PPV is about 50% [26]. This study is different from the previous retrospective analysis of the NIPT-PLUS test. Instead, the deletions detected by CMA were larger than 500 kb, fragments with repeats larger than 1000 kb were selected, and NIPT-PLUS detection was performed simultaneously to evaluate the detection effect of NIPT-PLUS on CNV. The data showed that the sensitivity of NIPT-PLUS to microdeletion and microduplication increased with the increase of CNV fragment size. For CNVs < 3 Mb, the detection rate of NIPT-PLUS was only 49.69%, of which 78.75% were suggested to be from the mother, which means that if the mother does not carry these small CNV fragments, it is very likely that they will be missed. Among the fragments > 10 M, NIPT-PLUS did not suggest that it was from the mother, but the missed detection rate reached 23.08%. On the one hand, the missed detection of this large deletion duplication may be related to the previously described false-negative and false-positive restricted placental mosaicism that causes SCA [34, 35]. On the other hand, this finding may be related to the low-depth sequencing of NIPT and limited data volume. Our data show that the detection rate of NIPT-PLUS for 5–10-Mb CNVs reached 100%, which is consistent with previously published data detection results [36]. In their study, Pei et al. [36] also showed that the PPV of CNVs < 10 Mb detected by NIPT was significantly higher than that of CNVs > 10 Mb. However, the detection rate of CNV detected by NIPT-PLUS in this study is more worthy of consideration for clinical application.

Similarly, we conducted statistical analyses on the detection rate of MMS commonly found in the clinical detection range of NIPT-PLUS. The data revealed that the detection rate of NIPT-PLUS for syndromes < 5 Mb, 1p36 microdeletion, cri-du-chat syndrome and chromosome 9p deletion (duplication) syndrome was 100%, while the detection rate for the most common DiGeorge syndrome reached 92.31%, and the detection rate for Angelman/Prader–Willi syndrome was 66.60%. Of course, these results need to be supported by large-sample data. However, the premise is that the size of the syndrome detected by CMA in this study was mainly 3–5 Mb, the detection rate of 15 cases of RACD syndrome (< 2 Mb) was 73.33%, and 8 of the 11 cases detected were suggestive of maternal origin. The detection rate of Williams–Beuren syndrome was the lowest, at about 33.3%. The current retrospective study showed that the PPV of NIPT-PLUS for MMS detection was 32% (CNVs ≥ 10 Mb) and 19% (CNVs < 10 Mb), and the PPV for common MMS was 93% (DiGeorge), 68% (22q11 0.22 microreplication), 75% (Angelman/Prader–Willi) and 50% (cri du chat) [23]. However, there are few reports on the detection rate of NIPT-PLUS for MMS detection. The detection rate and missed detection rate suggest the necessity and importance of NIPT-PLUS as a first-line clinical screening technique, with follow-up genetic counselling. It is suggested that although the PPV of CNV detection by NIPT-PLUS reached 50%, which was already higher than that of common prenatal screening techniques, it is also recognised that this technique has a missed detection rate for CNV and an optimal range for the detection of CNV fragment size. As mentioned above, we believe that NIPT-PLUS has better detection efficiency for CNVs > 5 Mb and is worthy of further clinical promotion, while it is not recommended for screening CNVs < 3 Mb due to its high missed detection rate. For CNVs between 3 and 5 Mb, the need to further inform clinics and pregnant women can be determined based on the pathogenic syndrome screened.

This study’s results contain cases that suggest that the results of CMA and NIPT-PLUS are not completely consistent. Among them, 4 cases of CMA suggested ROH and uniparental disomy, 3 of which were non-invasive and showed no abnormality, and 1 case of 16 ROH non-invasive suggested T16. In two other cases, the CMA suggested 47, XYY, and the NIPT-PLUS suggested 45, XO. The remaining 15 cases were mainly false positives for aneuploidy or large-fragment CNVs detected by NIPT-PLUS and false negatives for CNVs < 3 Mb that were missed. The influencing factors of this inconsistent result are related mainly to confined placental mosaicism (discussed above) and to the fact that most of the cffDNA detected by NIPT-PLUS was maternal fat metabolism DNA.

In the enrolled cases, NIPT-PLUS aneuploidy false negatives were also detected, which have also been reported in many studies. A recent review article suggests that the concentration of cffDNA in the peripheral blood of pregnant women, maternal characteristics, foetal–placental characteristics, experimental factors and calculation methods can all affect NIPT-PLUS test results, resulting in false negatives or false positives [37]. It seems that false positives are more acceptable than false negatives because the birth of an aneuploid foetus greatly impacts the family. In this study’s data, a total of 3 cases of false-negative aneuploidy were detected, and 1 pregnant woman underwent CMA testing due to NT thickening; the result indicated that it was 45, XO, and the NIPT-PLUS results both indicated that the risk was low. The post-induction placenta of the pregnant woman was obtained for verification, and the MLPA results of the placental subsurface and maternal surface showed a very low proportion of 45, XO mosaicism, while the umbilical cord showed complete 45, XO. One case was chimeric 45, XO due to the abnormal ultrasound detection of CMA, and both NIPT-PLUS tests were low risk. Another false-negative pregnant woman with trisomy 21 underwent CMA testing due to nasal bone deletion, and the result suggested chimeric T21. The chimeric ratio of FISH verification was 13.57% (19/140).

Non-invasive prenatal testing-PLUS suggests low risk. Hints of foetal or placental mosaicism and confined placental mosaicism are the most critical factors leading to false negatives in NIPT-PLUS. This is also consistent with the description in previous literature [36]. Similarly, NIPT-plus false positives are also common. Copy number variation false-positive results are due mostly to maternal chromosome imbalance, while false positives of aneuploidy are associated with foetal or placental mosaicism and restricted placental mosaicism, twins and single pregnancies, maternal benign or malignant fibroids and Z value data analysis [28, 38].

Few studies provide a detailed description of the detection range, sensitivity or miss rate of NIPT-PLUS. Our data suggest that NIPT-PLUS has a certain miss rate for the detection of chromosome aneuploidy and CNV, especially for small-fragment CNV. Additionally, few studies have conducted specific data analyses on the detection range of NIPT-PLUS, while our data were synchronised with CMA experiments according to the CNV size, obtaining NIPT-PLUS as the best detection range for first-line screening. These data show that NIPT-PLUS, as the main screening technology in first-line clinical practice, has very high sensitivity for aneuploidy detection. However, cytogenetic karyotype analysis may also be needed in some situations. The NIPT-PLUS technique has low sensitivity for CNV detection, especially at < 3 Mb, and it has a relatively high missed detection rate. Therefore, it is not recommended for clinical use as a first-line screening standard. For CNVs > 5 Mb, the sensitivity increases and the missed detection rate decreases. Accordingly, NIPT-PLUS can be performed appropriately in clinical testing, but it needs to be closely combined with comprehensive perinatal care, such as ultrasound, to avoid the occurrence of birth defects. In addition, the test results suggest that confined placental mosaicism and foetal mosaicism are the key factors causing false negatives in NIPT-PLUS. Maternal chromosomal abnormalities and confined placental mosaicism are key contributors to false positives, so appropriate genetic counselling is especially important for pregnant women before and after NIPT-PLUS testing.

This study has some limitations. First, the sample size of this study was small, which did not allow the sensitivity of NIPT-PLUS to be calculated for common syndromes. To obtain better statistical reliability, more studies with greater numbers of specimens are required. Second, there was a lack of familial genetic information. During the prenatal diagnoses, we did not conduct a family survey to determine whether the CNV was inherited from the mother in the foetus. In future studies, we will explore the clinical effects of NIPT-PLUS combined with other diagnostic methods.

## Data Availability

All data generated or analyzed during this study are included in this article.
